# HPV screening in the urine of transpeople - A prevalence study

**DOI:** 10.1016/j.eclinm.2022.101702

**Published:** 2022-10-12

**Authors:** Sophie Pils, Jana Mlakar, Mario Poljak, Grega Gimpelj Domjanič, Ulrike Kaufmann, Stephanie Springer, Andreas Salat, Eva Langthaler, Elmar A. Joura

**Affiliations:** a Department of Obstetrics and Gynaecology, Medical University of Vienna, Spitalgasse 23, 1090 Vienna, Austria; b Institute of Microbiology and Immunology, Faculty of Medicine, University of Ljubljana, Zaloška 4, 1000 Ljubljana, Slovenia; c Department of Surgery, Medical University of Vienna, Spitalgasse 23, 1090 Vienna, Austria; d Department of Pathology, Medical University of Vienna, Spitalgasse 23, 1090 Vienna, Austria.

**Keywords:** HPV prevalence, Transpeople, Transgender, FtM, MtF, Non binary

## Abstract

**Background:**

There is limited data on human papillomaviruses (HPV) prevalence in transpeople due to low acceptance rate of screening methods. HPV tests from self-collected urine are gender-neutral, have a high acceptance, and have a comparable accuracy in females to clinician-collected samples. The aim of this study was to evaluate both the HPV prevalence in the urine in a large cohort of 200 transpeople with common risk profiles and the acceptability of such screening method.

**Methods:**

The study was conducted at the outpatient clinic for transpeople at the Department of Obstetrics and Gynaecology, Medical University of Vienna, Austria. 200 transpeople have been enrolled between May and October 2021. Inclusion criteria were gender identity dysphoria, age over 18 years, and adequate language skills.

Subjects were asked to answer a survey concerning gender identity, established risk factors for HPV infections as well as their preference regarding urine or provider-collected cytology-/HPV-based screening, and to provide a urine sample. Five patients not able to provide urine were excluded. HPV genotyping was performed using a validated multiplex real-time PCR assay, which simultaneously detects 28 HPV genotypes. This trial is registered at ClinicalTrials.gov, NCT04864951.

**Findings:**

Overall HPV positivity was 19·0% (37/195), 24·2% in female to male, 11·8% in male to female, 26·3% in genderqueer/non binary/other subjects, 27·9% in subjects currently having a cervix, and 26·0% in subjects born with cervix. Independent of gender reassignment surgery, being born with a cervix was associated with a higher risk of HPV infections (*p* = 0·008), yet 42·3% (44/104) have never attended cervical cancer screening. Overall, 79·0% (154/195) of transpeople would prefer urine HPV tests to provider-collected HPV screening.

**Interpretation:**

HPV testing in self-collected urine samples provides a unique opportunity for screening of this hard-to-reach population and should be evaluated in further studies.

**Funding:**

None.

**Research in context**

**Evidence before the study**

At the time of trial design (October 2020), we searched PubMed for all article types using search terms: “HPV prevalence” + “transgender” + “transmen” + “transfemale” with no restrictions on date. Non-English/French/German articles were excluded. We excluded all studies that focused on HPV vaccination coverage and not HPV infection rates, and studies that did not distinguish between transvestites, men who have sex with men, or transgender women. No meta-analysis has ever been conducted, reviews point out the lack of data.

Persistent infections with high-risk genotypes of human papillomaviruses (HPV) can cause precancerous lesions and lead to invasive cancer if untreated. There are only a few publications about HPV prevalence in transpeople, especially with common risk profiles e.g. non sex-workers.

**Added value of this study**

We present a non-invasive HPV screening method with high acceptance in a vulnerable population.

This large study describes an HPV prevalence of 19·0% (37/195) in self-collected urine of transpeople with common risk profiles. Furthermore, detailed data for female to male, male to female or genderqueer/non binary/other non-binary transpeople with current or former presence of a cervix is presented.

Independent of gender reassignment surgery, being born with a cervix was associated with a higher risk of HPV infections (p = 0·008), yet 42·3% (44/104) have never attended cervical cancer screening. Therefore, 379·0% (154/195) of transpeople would prefer urine HPV tests to provider-collected cytology-based screening.

**Implications of all the available evidence**

HPV testing of self-collected urine may provide a unique opportunity for screening of this hard-to-reach population and should be evaluated in further studies.

Alt-text: Unlabelled box

## Introduction

Persistent infections with high-risk genotypes of human papillomaviruses (HPV) can cause precancerous lesions and lead to cancer if untreated. Approximately 99% of all cervical cancers, 88% of anal cancer, 70% of vaginal cancer, 25% of vulvar cancer, 30% of penile cancer, and up to 22% of oropharyngeal cancers are due to persistent HPV infections.^1^, ^2^, ^3^ Furthermore, neovaginas are also at risk.^4^ In addition, infections with low-risk HPV genotypes cause virtually all anogenital warts and laryngeal papillomas; HPV 6 also has moderate oncogenic potential and has been shown to cause penile cancer.^5^ HPV screening is the gold standard to reduce incidence and mortality of cervical cancer and other HPV-related diseases.^6^

There is limited literature data on HPV prevalence in transpeople with common risk profiles and obtained mostly in small cohorts, but it is generally assumed that it is as high or even higher than in cispeople ranging from 16·0% (21/131) to 21·4% (3/14) in female to male (FtM), 11·5% (6/52) in neovaginal, and from 52·4% to 88·6% (11/21; 9/13; 39/44) in anal samples in male to female (MtF).^7^, ^8^, ^9^, ^10^, ^11^

Independently of national health systems or screening strategies, acceptance of cytology- or HPV-based screening is lower for transmen than for ciswomen especially because of the need for genital examination.^12^, ^13^, ^14^ Furthermore, childhood sexual abuse has been described in a fifth of transpeople, being the highest in FtM.^15^ Consequently, transmen prefer self-collected vaginal swabs to provider-collected cervical swabs.^7^

Devices for self-collected urine are licensed gender-neutral, have a high acceptance, and the same HPV testing accuracy as clinician-collected samples in people with a cervix.^16^^,^^17^

Although a penile swab from the external urethral meatus, the glans, the coronal sulcus, and the shaft is currently the gold standard for research purposes, most previous MtF studies used anal sampling even in presence of a penis.^8^, ^9^, ^10^, ^11^^,^^18^ Due to the discomfort felt by transpeople to their anatomical sex, penile swabbing is avoided in MtFs even though this is potentially associated with lower analytical sensitivity of the HPV testing.

The aim of this study was to evaluate the HPV prevalence in the urine of a large cohort of 200 transpeople and the acceptance of this HPV-based screening.

To the best of our knowledge, this is the largest study on HPV prevalence in transpeople with common risk profiles, e.g. non sex-workers, conducted so far. Additionally, it is the first study to determine HPV in urine of transpeople.

## Methods

The study was conducted at the outpatient clinic for transpeople at the Department of Obstetrics and Gynaecology, Medical University of Vienna, Austria. All 203 transpeople who attended the outpatient clinic for hormonal treatment monitoring on a scheduled study day were assessed for eligibility between May and October 2021, three declined to participate, 200 were included in this study. Inclusion criteria were gender identity dysphoria, age over 18 years, and adequate language skills. All eligible patients were invited to participate in the study regardless of gender reassignment surgery. After the allocation number was assigned, subjects were asked to fill out a survey with questions concerning gender identity, sexual orientation, number of sexual partners, history of cytology- or HPV-based screening, HPV vaccine status, smoking, preference regarding urine or provider-collected cytology-/HPV-based screening, and to provide a urine sample (see online supplementary text 1). Two informed consent forms, one survey and the prefilled collector tube for self-collected urine were labelled with the subject´s allocation number. All subjects received the results of the HPV test by mail up to the 3 weeks after examination, and, in the case of an HPV positive result, further examinations and counselling at the Medical University of Vienna, outside the study settings, were offered.

Out of 200 eligible patients, five patients were not able to provide a urine sample during the visit and were excluded from the study.

Urine samples were collected using Colli-Pee FV-5000® series prefilled with 3·4 millilitres (ml) Urine Conservation Medium (CE Ref N00176, Novosanis, Wijnegem, Belgium) allowing a collection of 9-12 ml first-void urine. This collection method has been used for HPV prevalence assessments to monitor the impact of HPV vaccination in Bhutan and Rwanda and is currently used in an HPV prevalence study in Thailand.^19^^,^^20^

The labelled samples were shipped to the Institute of Microbiology and Immunology, Faculty of Medicine, University of Ljubljana, Slovenia for HPV testing.

After arrival in the laboratory urine samples were stored at 4°C and processed within 3 days. Briefly, the urine samples were thoroughly vortexed, 5 ml of the urine was transferred to a 15-ml tube, the remaining urine was aliquoted into 2-ml microcentrifuge tubes, and stored at −80°C. Cells were pelleted by centrifugation at 800 rcf for 10 min and all but approximately 200 µl of the supernatant was discarded. Cells were resuspended in the remaining supernatant solution. DNA was extracted from 200 µl of the sample using the QIAamp DNA Mini Kit (Qiagen, Hilden, Germany). The DNA was eluted in 50 µl of buffer AE into a 1·5 ml microcentrifuge tube. If not processed immediately, the isolated DNA was stored at −20°C.

HPV detection and genotyping was performed using the widely used and well validated multiplex real-time PCR assay, Anyplex II HPV 28 Detection (Seegene, Seoul, South Korea), which simultaneously detects 28 HPV genotypes (6, 11, 16, 18, 26, 31, 33, 35, 39, 40, 42, 43, 44, 45, 51, 52, 53, 54, 56, 58, 59, 61, 66, 68, 69, 70, 73, 82) and an internal control. Five µl of isolate was used in two wells of the run. One negative control and three positive controls from the kit were used in each PCR run. Analysis of the results was performed automatically using Seegene Viewer software according to the manufacturer's instructions. In the Ljubljana laboratory, Anyplex II HPV 28 Detection kit demonstrated 100% proficiency (100% sensitivity and 100% specificity) when testing 2019 and 2021 HPV Labnet international genotyping proficiency panels consisting of 44 blinded specimens and prepared by WHO International HPV Reference Center (Karolinska Institutet, Stockholm, Sweden).^21^

### Ethics statement

Approval for this study was obtained from the Ethical Review Board of the Medical University of Vienna (IRB 1186/2021). This study follows the Consolidated Standards of Reporting Trials (CONSORT) reporting guideline. The study was registered on clinicaltrials.gov (NCT04864951). All patients provided written, informed consent.

### Outcome measures

The primary outcome measure was HPV prevalence in the urine of transpeople. Secondary, known risk factors for HPV infection and persistence were analysed. Furthermore, acceptance of HPV-based screening using urine samples was evaluated.

### Statistical analysis

Nominal variables were reported as numbers and frequencies, and continuous variables as medians and interquartile ranges. Nominal variables between groups were compared using the Chi square test, and continuous variables were compared between groups using the Mann-Whitney-U-Test as they were non-normally distributed according to a Kolmogorov-Smirnov Test. All statistical tests were 2-sided. Significant parameters were entered in a multivariate logistic regression model. All analyses were performed using SPSS statistics for Windows, version 27·0 (SPSS Inc., Chicago, IL, USA), and p-values < 0·05 were considered statistically significant.

### Role of funding source

There was no funding. All authors had full access to the data and were responsible for the decision to submit for publication.

## Results

**Figure 1:**
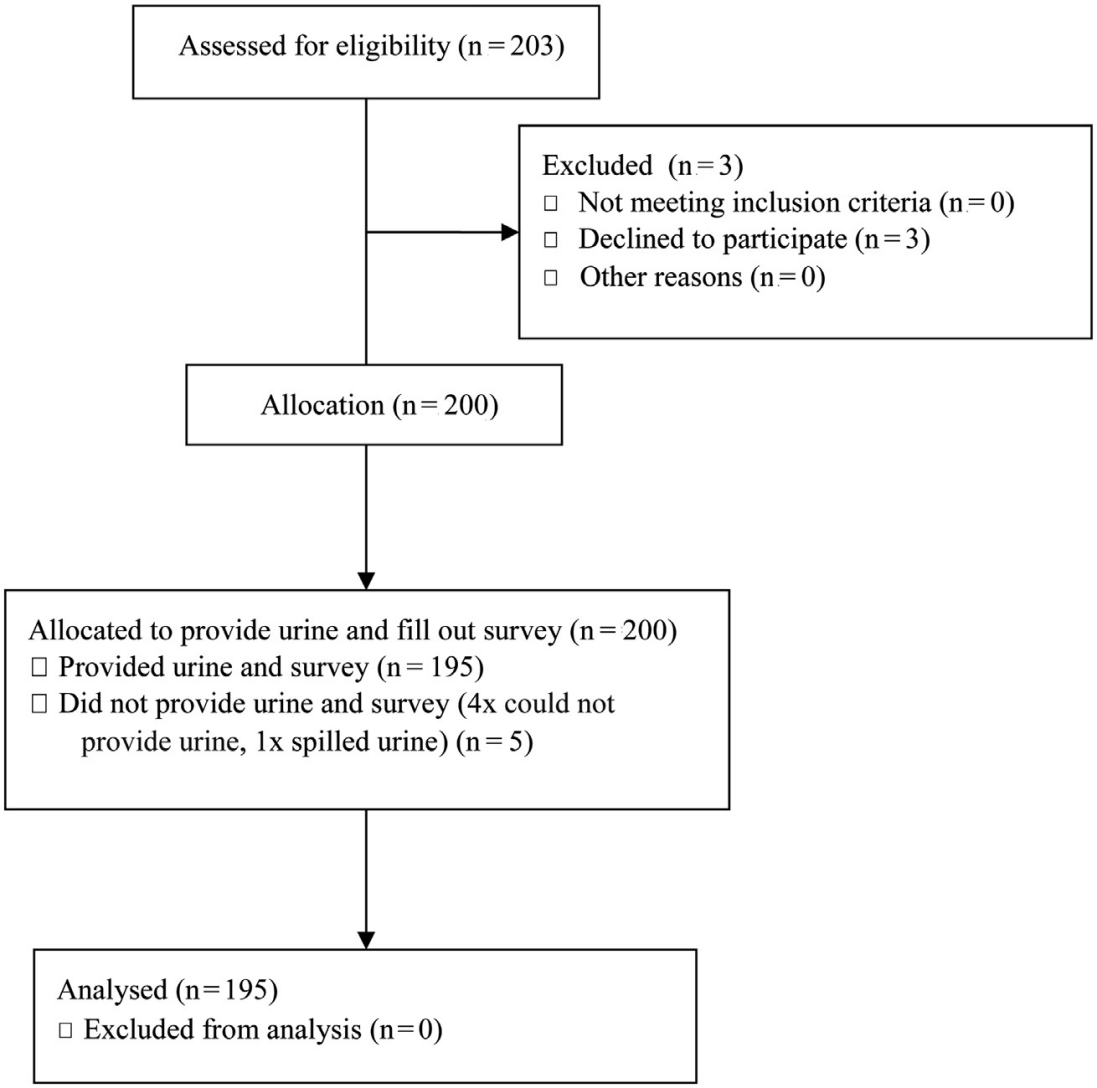
**Flow diagram**.

203 transpeople were invited to participate in the study and three of them declined, therefore the willingness to participate in this study was 98·5% (200/203). A total of 200 transpeople were enrolled, but five individuals had to be excluded due to missing urine samples (four subjects could not provide urine although water was offered; one subject spilled some of the urine when removing the tube from the housing; Figure 1). Basic patient characteristics are provided in Table 1.

**Table 1:** Basic characteristics, *n* = 195.

Age^1^		28 (23–36)
Gender identity^2^	FtM	91 (46·7)
	MtF	85 (43·6)
	Genderqueer/non binary	18 (9·2)
	Another gender identity	1 (0·5)
Sexual orientation^2^	homosexual	34 (17·4)
	heterosexual	62 (31·8)
	bisexual/ pansexual	76 (39·0)
	asexual	8 (4·1)
	Other/did not want to answer	15 (7·7)
HIV positivity^2^		2 (1·0)
Hormone use^2^		173 (88·7)
	Testosterone	90 (46·2)
	Estrogen	81 (41·5)
	Progesterone only	2 (1·0)
Antidepressants/antipsychotics^2^		44 (22·6)
Transgender surgeries^2^		42 (21·5)
	Hysterectomy and oophorectomy	18 (9·2)
	Penoid	1 (0·5)
	Orchiectomy and neovagina	23 (11·8)
HPV vaccinated^2^	Yes	20 (10·3)
	Age at vaccination	16.5 (14·0–21·7)
Cigarette smoking^2^	No	138 (70·8)
	1–5 a day	12 (6·2)
	5–10 a day	14 (7·2)
	>10 a day	31 (15·9)
Number of sexual partners in lifetime^1^		4 (1–10)
Number of sexual partners past 12 months^1^		1 (0–1)

Abbreviations used: n, number; FtM, female to male; MtF, male to female; HIV, human immunodeficiency virus; HPV, human papillomavirus.

1 median (interquartile range) or

2 number (frequencies).

Combined prevalence for any of the 28 targeted HPV genotypes by the clinically validated Anyplex II HPV 28 Detection (Seegene, Seoul, South Korea) was 19·0% (37/195), 24·2% (22/91) in FtM, 11·8% (10/85) in MtF, 26·3% (5/19) in genderqueer/non binary/other subjects, 27·9% (24/86) in subjects currently having a cervix, and 26·0% (27/104) in subjects born with cervix (Table 2). All samples could be assayed. There were no statistically significant differences regarding prevalence of HPV infection regarding age or gender identity, while well-established risk factors for HPV infections like number of sexual partners, and cigarette smoking were significant. Only smoking and being born with a cervix remained statistically significant after multivariate analysis (Table 3). Detailed data of HPV positive subjects are provided in Table 4.

**Table 2:** Key findings from previous HPV prevalence studies in transpeople published in peer-reviewed literature in comparison to findings of the present study.

	*n*	HPV positivity in FtM	HPV positivity in MtF	HPV positivity in genderqueer/non binary/other	Site	HPV Assay used	Research Objective
Reisner et al.^5^	131	16·0% (21/131)			Cervicovaginal	Digene Hybrid Capture II (Qiagen)	Self- versus provider-collected swabs
Loverro et al.^6^	35	21·4% (3/14)	52·4% (11/21)		Anal (Oral, vaginal, cervicovaginal, penile not presented)	Linear Array Genotyping Assay (Roche)	HPV prevalence
Van der Sluis et al.^7^	52		11·5% (6/52)		Neovaginal	HPV-Risk assay (Self-Screen BV)	HPV prevalence
Cranston et al.^8^	13		69·2% (9/13)		Anal	Papillocheck (Greiner)	HPV prevalence
Singh et al.^9^	44		88·6% (39/44)		Anal (oral, serum not presented)	Linear Array Genotyping Assay (Roche)	HPV prevalence
Brown et al.^17^	68		95·6% (65/68)		Anal, penile	Linear Array Genotyping Assay (Roche)	HPV and HIV prevalence in a cohort in which a majority received money or favours for sex
Jalil et al. ^18^	272		77·9% (212/272)		Anal	Papillocheck (Greiner)	HPV prevalence in a cohort with a large sex-worker rate
Pils et al.	195	24·2% (22/91)	11·8% (10/85)	26·3% (5/19)	Urine	Anyplex II HPV 28 Detection (Seegene)	HPV prevalence

Abbreviations used: n, number; FtM, female to male; MtF, male to female; HPV, human papillomavirus; HIV, human immunodeficiency virus.

Digene Hybrid Capture II (Qiagen, Gaithersburg, MD, USA): HPV genotypes 16, 18, 31, 33, 35, 39, 45, 51, 52, 56, 58, 59, 68.

Linear Array HPV Genotyping Test (Roche Molecular Systems, Branchburg, NJ, USA): HPV genotypes 6, 11, 16, 18, 26, 31, 33, 35, 39, 40, 42, 45, 51, 52, 53, 54, 55, 56, 58, 59, 61, 62, 64, 66, 67, 68, 69, 70, 71, 72, 73, 81, 82, 83, 84, 89, IS39.

HPV-Risk assay (Self-Screen BV, Amsterdam, Netherlands): HPV genotypes 16, 18, 31, 33, 35, 39, 45, 51, 52, 56, 58, 59, 66, 67, 68.

Papillocheck (Greiner Bio-One, Frickenhausen, Germany): HPV genotypes 6, 11, 40, 42, 43, 44, 16, 18, 31, 33, 35, 39, 45, 51, 52, 53, 56, 58, 59, 66, 68, 70, 73, 82.

Anyplex II HPV 28 Detection (Seegene, Seoul, South Korea): HPV genotypes 6, 11, 16, 18, 26, 31, 33, 35, 39, 40, 42, 43, 44, 45, 51, 52, 53, 54, 56, 58, 59, 61, 66, 68, 69, 70, 73, 82.

**Table 3:** Comparison between HPV positive and HPV negative transpeople, *n* = 195.

			HPV positive *n* = 37 (19·0)	HPV negative *n* = 158 (81·0)	Univariate analysis	Multivariate analysis
					*p*	*p*
Age^1^			29 (24–38)	27 (23–36)	0·311	
Gender identity ²		FtM	22 (59·5)	69 (43·7)	0·132	
		MtF	10 (27·0)	75 (47·5)		
		Genderqueer/non binary	5 (13·5)	13 (8·2)		
		Another gender identity	0 (0·0)	1 (0·6)		
Born with a cervix²			27 (73·0)	77 (48·7)	*0·008*	*0·011*
Number of sexual partners in lifetime^1^			10 (5–20)	3 (1–8)	*<0*·*001*	*0·743*
Number of sexual partners past 12 months^1^			1 (1–2)	1 (0–1)	*<0*·*001*	*0·386*
Cigarette smoking ²		No	16 (43·2)	122 (72·2)	*<0*·*001*	*0·008*
		1–5 a day	7 (18·9)	5 (3·2)		
		5–10 a day	5 (13·5)	9 (5·7)		
		>10 a day	9 (24·3)	22 (13·9)		
HPV vaccinated ²			7 (18·9)	13 (8·2)	0·054	
Age at vaccination (years)^1^			15 (13-24)	18 (14-19)	0·905	

Data are presented as ¹ median (interquartile range) or ² number (frequencies); significant p-values are provided in italics.

Abbreviations used: n, number; FtM, female to male; MtF, male to female; HPV, human papillomavirus.

**Table 4:** Gender identity, presence of cervix or neovagina, distribution of HPV genotypes and follow-up medical procedures in 37 HPV positive subjects (preventable HPV types are in bold).

Gender identity	Cervix	Neovagina	HPV	Follow-up	Dysplasia	Procedure, histology
FtM	x		**16**	no		Hysterectomy, no dysplasia
FtM	x		**16**	yes	yes	LLETZ, HSIL
FtM	x		53, 66	no		Hysterectomy, no dysplasia
FtM	x		59	no		
FtM	x		51	yes	no	
FtM	x		**6**	yes	yes	
FtM	x		43, 56	yes	no	
FtM	x		61, 73	yes	no	
FtM	x		42, 66	yes	no	
FtM	x		**6, 16**, 42, **45, 52**, 61, 66, 70	yes	no	
FtM	x		42, 51, 53, 70	no		
FtM	x		44	no		
FtM	x		**6**, 42, 66	no		
FtM	x		**11,** 54, 70	yes	no	
FtM	x		56	yes	no	
FtM	x		54	yes	no	
FtM	x		**31**	no		Hysterectomy, no dysplasia
FtM	x		**6**, 54	yes	no	
FtM	x		54	no		
FtM			**33,** 59	no		
FtM			54	no		
FtM			**31, 58**	no		
Genderqueer/non binary/other	x		44	yes	no	
Genderqueer/non binary/other	x		42**, 45,** 51	yes	no	
Genderqueer/non binary/other	x		42	yes	no	
Genderqueer/non binary/other	x		**31,** 39, 51, 53	yes	yes	LLETZ, HSIL
Genderqueer/non binary/other	x		40, 53	no		
MtF		x	61	yes	no	
MtF		x	43	yes	no	
MtF		x	61	yes	no	
MtF		x	39, 43, 51, 53	no		
MtF		x	61	yes	no	
MtF			**6,** 59	yes	condyloma	Imiquimod
MtF			54	yes	no	
MtF			59	no		
MtF			**31**	yes	yes	
MtF			42	no	no	

Abbreviations used: FtM, female to male; MtF, male to female; HPV, human papillomavirus; LLETZ, large loop excision of the transformation zone; HSIL, high grade intraepithelial lesion.

Independently of gender reassignment surgery, being born with a cervix was associated with a higher risk of HPV infection (*p* = 0·008), yet 42·3% (44/104) have never attended cervical cancer screening. Subjects who underwent screening at least once described that they experienced the pain during examination of 6 grade (4-8) on the visual analogue scale (VAS; range 0-10, where 0 means no pain and 10 means pain is intolerable) and would therefore prefer an urine HPV test to cytology-based screening in 88·3% (53/60), 7 subjects stated that they did not know, no one would prefer cytology-based screening. In addition, subjects with a neovagina underwent cytology-based screening in 21·7% (5/23) and would prefer urine HPV test to cytology-based screening in 80·0% (4/5). Overall, 79·0% (154/195) would prefer urine HPV tests to provider-collected cytology-based screening, 21·0% (41/195) did not know, no one preferred a cytology-based screening.

In case of HPV positivity, a clinical examination and counselling was offered on a voluntary basis. 22 out of 37 subjects accepted the offer. Out of these, two cases (9%) of cervical high grade squamous intraepithelial lesion (HSIL) were diagnosed and treated with a large loop excision of the transformation zone (LLETZ); another one was treated with Imiquimod (three times a week for 16 weeks; Aldara® 5% cream; MEDA Pharma GmbH & Co. KG; Bad Homburg) due to condyloma. Three instead underwent gender reassignment surgery with hysterectomy with bilateral salpingo-oophorectomy in the six months following the study, no dysplasia was found in the surgical specimen (Table 4).

## Discussion

Combined HPV prevalence in the urine samples obtained from 195 transpeople in this study was 19·0% for the 28 targeted HPV genotypes. HPV prevalence in subjects currently having a cervix in this study was higher (27·9%; 24/86) than previously described by Reisner et al. (16·0%; 21/131).^7^ However, if only the oncogenic HPV types 16, 18, 31, 33, 35, 39, 45, 51, 52, 56, 58, 59, and 68 commonly targeted in both studies are taken into account, prevalence is almost identical with 16·3% (14/86) (Table 2), and thus also comparable to the HPV prevalence of ciswomen, which ranges from 10·6% to 21·8%, depending on age.^22^

HPV prevalence in MtF was 11·8% (10/85) which is comparable to 11·5% (6/52) HPV positivity in neovaginal HPV sampling described by Van der Sluis et al.^9^ When comparing urine to anal HPV sampling, prevalence is considerably lower in urine samples. However, it should be noted that the studies by Loverro et al. (11/21 HPV positive) and Cranston et al. (9/13 HPV positive) had a small number of subjects.^8^^,^^10^ Furthermore, in the study of Singh et al. (39/44 HPV positive) at least 26·5% of the subjects were HIV positive.^11^ In studies where the majority of MtF subjects were sex workers, HPV prevalence was 77·9% (212/272) to 95·6% (65/68).^23^^,^^24^ In cismen, HPV prevalence from swabs from the penis and scrotum is 36·0% in men having sex with men (MSM), 50·8% in men having sex with women and men (MSWM), 42·1% in men having sex with women (MSW). However, it should be noted that 27·7% (23/83) of MtF had ten or more lifetime sexual partners, compared to 55·6% and 43·0% for MSM and MSW, respectively.^25^

Nevertheless, while the analytical sensitivity of HPV testing in urine from a penile urethra has substantially increased and study protocols were adopted, it still appears to be inferior to swabs.^18^^,^^26^ Therefore, the lower HPV prevalence recorded in this study may potentially be due to sampling method. However, HPV testing in urine is non-invasive and the same device can be used regardless of anatomical sex.

Regarding the acceptance of study participation of 98·5% (200/203), only Loverrro et al. included subjects during the visit of the outpatient clinic for hormonal treatment monitoring and reported a participation rate of 87·5% (35/40).^8^ Sample collection rate in our study of 97·5% (195/200) is comparable to Reisner et al. who reported that 93·3% (140/150) individuals were willing to provide the samples, although $100 incentives upon completion of the study activities were provided.^7^

Pain during genital examination is considerably higher in FtM (6 (4-8) on the VAS) than in ciswomen whose pain during speculum insertion was described with less than 2 on the VAS (1·7 (0-7·5)). For comparison, pain during speculum insertion and biopsy for HPV-associated lesions was described with 3·0 (0-8·5) on the VAS.^27^ This is in concordance with Peitzmeier et al, who summarized that in addition to psychological reasons, speculum insertion is more painful due to vaginal atrophy from long-term hormonal therapy.^28^

Childhood sexual abuse was described by 19% in transpeople, being the highest in FtM.^15^ A HPV prevalence of 17% in girls after sexual abuse had been described previously.^29^ Furthermore, compliance with cytology-based screening is lower after sexual assault.^30^ Moreover, independently of a history of abuse, acceptance of cytology- or HPV-based screening, especially due to the genital examination, is lower for transmen than for ciswomen.^12^, ^13^, ^14^ It has already been shown that HPV testing on self-collected samples has a high acceptance rate, but HPV testing in urine, being thoroughly non-invasive, goes one step further and has been demonstrated to be a reliable sampling method in individuals with a cervix.^7^^,^^16^^,^^17^ In this study, not only was the willingness to participate impressively high (only 3 out of 203 invited subjects declined to participate), but also the subjects’ appreciation that their medical problems were being recognized was remarkable.

The reported HPV vaccination rate of 10·3% in the study population is low. Even if we analyse only those subjects who had had the possibility to be vaccinated against HPV since 2014, when Austria implemented its gender-neutral children's HPV vaccination program, vaccine uptake rate is 17·4%.

The combination of a rate of childhood sexual abuse in transpeople that is twice as high as in cispeople, a modest HPV vaccination rate amongst transpeople, and a low acceptance of cytology- or HPV-based screening puts transpeople at particularly high-risk for HPV-related cancers.

Limitations of this study are that childhood sexual abuse was not evaluated and the presence of HPV was only analysed in the urine and thus not compared to the sample type used in previously studies, e.g., provider-collected cervical, penile or anal swabs or self-collected swabs for hrHPV DNA detection. Furthermore, in Austria, transpeople must submit a clinical-psychological, a psychiatric, and a psychotherapeutic statement before starting hormonal treatment as well as before gender reassignment surgery. All but one subject had public health insurance. We investigated a Central European sample in a high resource country, therefore findings from this single centre study may not be generalised for different populations around the world. Moreover, due to the study setting, a self-reporting bias may have occurred, whereby especially the relevant questions regarding the number of sexual partners, pain during smear collection, preference for urine or smear collection, and nicotine consumption cannot be verified in the medical records. In addition, HPV testing protocol used has neither been validated from urine from a penile urethra nor in individuals without a cervix.

The recommendation made for victims of sexual abuse to start HPV screening at age 18 and to provide awareness programs with generous access to HPV vaccination, is equally applicable to transpeople.^31^ Furthermore, self-sampling HPV screening in urine of people born with a cervix could greatly facilitate the acceptance of the screening program by this vulnerable group, for people born with a penis further studies have to be done regarding sensitivity of urine analysis.

In conclusion, this is the largest study on HPV prevalence in transpeople with common risk profiles conducted so far and the first to determine HPV in the urine of transpeople. HPV infections are common and we identified persons at risk. Our data show that HPV testing of self-collected urine has a high acceptance rate in transpeople. HPV testing of self-collected urine may provide a unique opportunity for screening of this hard-to-reach population and should be evaluated in further studies.

## Contributors

Sophie Pils: the idea for the article, the literature search, the project's and the manuscript's conception and design, acquisition of data, statistical analyses, drafting the article and revising it for intellectual content, final approval of the version to be published.

Jana Mlakar: the project's and the manuscript's conception and design, laboratory analysis, final approval of the version to be published.

Mario Poljak: the project's and the manuscript's conception and design, the literature search, laboratory analysis, drafting the article and revising it for intellectual content, final approval of the version to be published.

Grega Gimpelj Domjanič: the project's and the manuscript's conception and design, acquisition of data, final approval of the version to be published.

Ulrike Kaufmann: the project's and the manuscript's conception and design, acquisition of data, drafting the article and revising it for intellectual content, final approval of the version to be published.

Stephanie Springer: the project's and the manuscript's conception and design, acquisition of data, statistical analyses, drafting the article and revising it for intellectual content, final approval of the version to be published.

Andreas Salat: acquisition of data, drafting the article and revising it for intellectual content, final approval of the version to be published.

Eva Langthaler: acquisition of data, drafting the article and revising it for intellectual content, final approval of the version to be published.

Elmar A. Joura: the idea for the article, the literature search, the project's and the manuscript's conception and design, acquisition of data, drafting the article and revising it for intellectual content, final approval of the version to be published.

## Data sharing statement

Anonymized data will be shared with a signed data access agreement with publication on request (Sophie.pils@meduniwien.ac.at). Study protocol will be shared with publication on request (Sophie.pils@meduniwien.ac.at).

## Declaration of interests

Elmar A. Joura reports advisory board fees, lecture fees and grants from Merck, and advisory board fees and lecture fees from Roche Diagnostics all outside of this study. Sophie Pils reports lecture fees from Merck outside of this study. Jana Mlakar, Mario Poljak, Grega Gimpelj Domjanič, Ulrike Kaufmann, Stephanie Springer, Andreas Salat, and Eva Langthaler report no conflict of interest.

## Appendix Supplementary materials

Image, application 1
