# Supplementary material for: HPV screening in the urine of transpeople - A prevalence study
Source: eClinicalMedicine. 2022 Oct 12;54:101702. doi: 10.1016/j.eclinm.2022.101702 (PMC9574404; doi:10.1016/j.eclinm.2022.101702)
Supplement: Supplementary file 1 [file mmc1.pdf]

UNIVERSITÄTSKLINIK FÜR FRAUENHEILKUNDE MEDIZINISCHE UNIVERSITÄT WIEN

# HPV screening in the urine of transpeople

Allocation Number: ________________
Date: _____________

1. Age: ____
2. Gender identity:
   ☐ female to male (FtM)
   ☐ male to female (MtF)
   ☐ genderqueer/non binary
   ☐ other genderidentity

3. Sexual orientation:
   ☐ homosexual
   ☐ heterosexual
   ☐ bisexuall / pansexual
   ☐ asexual
   ☐ not clear or do not want to answer

4. Number of sexual partners throughout life: ____
5. Number of sexual partners in the last 12 months: ____
6. Current medication: ____________________________________________________________
_____________________________________________________________________________
_____________________________________________________________________________

7. Have you already had gender reassignment surgery?
   ☐ yes
   ☐ no

   If yes:
   ☐ ovaries removed
   ☐ uterus with cervix removed
   ☐ uterus removed without cervix
   ☐ penoid
   ☐ testicles removed
   ☐ penis removed
   ☐ neovagina

8. When did you have your last PAP smear?
   ☐ less than a year ago
   ☐ 1-3 years ago
   ☐ over 3 years ago
   ☐ never

9. If you have ever had a PAP smear, how did you feel about the collection?

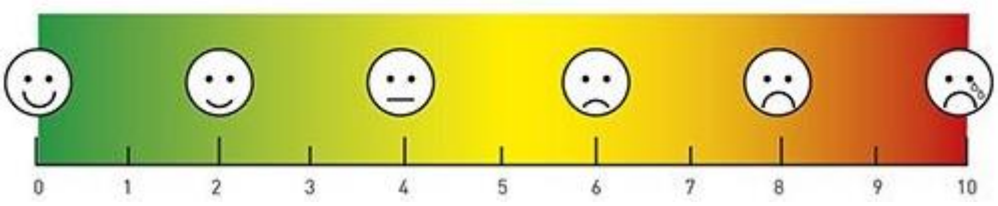

10. Have you ever had an abnormal Pap smear?
☐ yes
☐ no
☐ I do not know
☐ I have never had a pap smear

11. When did you have your last HPV test?
☐ in the past 3 years
☐ over 3 years ago
☐ never

12. If you have ever had an HPV test, how did you feel about the collection?

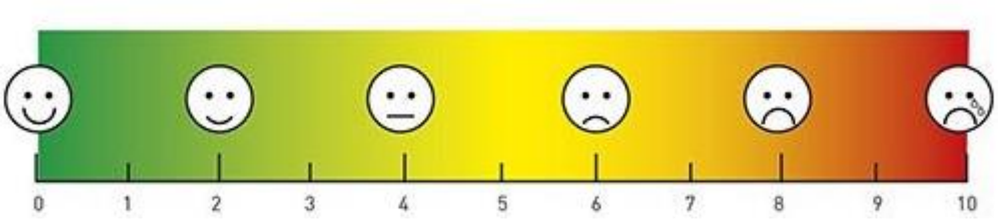


13. Have you ever had a positive HPV test?
☐ yes
☐ no
☐ I do not know
☐ I have never had a HPV test

14. Would you prefer a urine HPV test to a Pap smear?
☐ yes
☐ no
☐ I do not know

15. Would you prefer an HPV test in urine to an HPV smear test?
☐ yes
☐ no
☐ I do not know

16. Are you vaccinated against HPV?
☐ yes
☐ no
☐ I do not know

17. If you were vaccinated against HPV, how old were you? ______

18. How many cigarettes do you smoke per day?
☐ I do not smoke
☐ 1-5 per day
☐ 5-10 per day
☐ over 10 per day

Thank you for your precious time!
